# Perspectives on Probiotics and Bronchopulmonary Dysplasia

**DOI:** 10.3389/fped.2020.570247

**Published:** 2020-10-23

**Authors:** Kun Yang, Wenbin Dong

**Affiliations:** Department of Newborn Medicine, The Affiliated Hospital of Southwest Medical University, Luzhou, China

**Keywords:** bronchopulmonary dysplasia, probiotics, gut-lung axis, microbiota dysbiosis, inflammation

## Abstract

Bronchopulmonary dysplasia (BPD) is a chronic respiratory disease of preterm infants, associated with high morbidity and hospitalization expenses. With the revolutionary advances in microbiological analysis technology, increasing evidence indicates that children with BPD are affected by lung microbiota dysbiosis, which may be related to the illness occurrence and progression. However, dysbiosis treatment in BPD patients has not been fully investigated. Probiotics are living microorganisms known to improve human health for their anti-inflammatory and anti-tumor effects, and particularly by balancing gut microbiota composition, which promotes gut-lung axis recovery. The aim of the present review is to examine current evidence of lung microbiota dysbiosis and explore potential applications of probiotics in BPD, which may provide new insights into treatment strategies of this disease.

## Introduction

**Figure 1:**
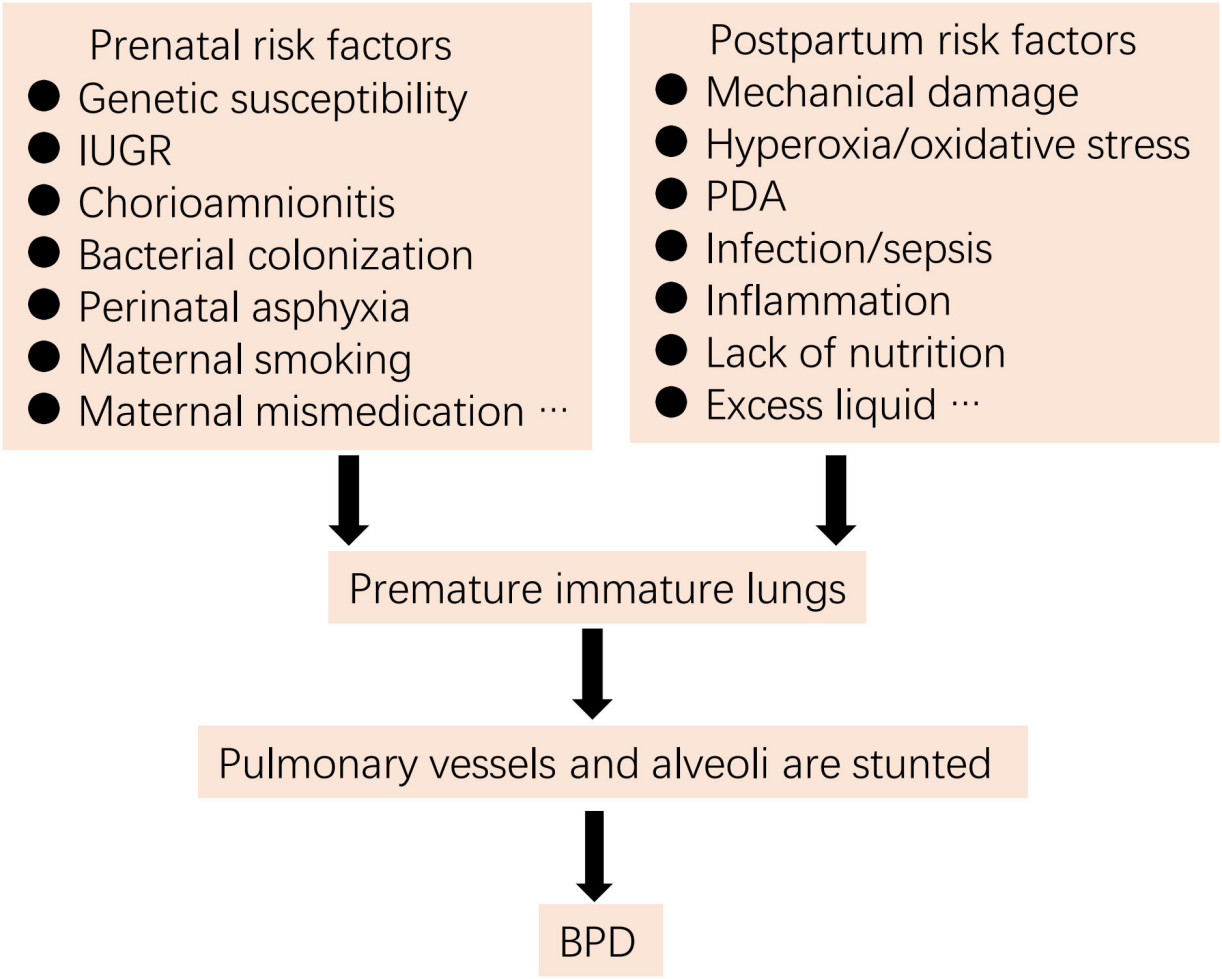
Risk factors for BPD (IUGR, intrauterine growth restriction; PDA, patent ductus arteriosus).

For more than 50 years, the definition, epidemiology, pathophysiology, and pathogenesis of BPD have been continuously updated (1–4). BPD was initially proposed by Northway and colleagues (5) in 1967 as a lung injury in preterm neonates due to mechanical ventilation and oxygen poisoning. However, current pathogenesis is more complex, involving exposure of infants to one or multiple pre- and/or post-natal high-risk events associated with lung immaturity, perinatal infection, inflammation, and altered blood vessel development (1, 2, 4, 6) (Figure 1).

The term probiotics derived from a Greek meaning “for life” (7), was coined by Lilly and Stillwell (8) in 1965, and described microorganisms with potential to release growth-promoting factors. In 2002, the FAO/WHO defined probiotics as “*live microorganisms which when administered in adequate amounts confer a health benefit on the host*,” but in 2013, The International Scientific Association for Probiotics and Prebiotics reached a consensus on modifying the concept to “*live microorganisms that, when administered in adequate amounts, confer a health benefit on the host*,” including microbial species that have been shown to confer health benefits in controlled studies, and new commensal and consortium strains from human samples, with evidence of safety and efficacy (9). At present, commonly used probiotics derive from the *Bifidobacterium* and *Lactobacillus* genus.

In recent years, a large body of evidence has related BPD to microbiota dysbiosis, however, therapy approaches have rarely been discussed or investigated. Therefore, this review explores new frontiers of probiotics to ameliorate BPD based on microbiota recovery.

## Lung Microbiota and BPD

Microorganisms living in a given environment constitute a microbiota, whereas a microbiome relates to microbial genome, metabolism, and growth surroundings (10). It then becomes relevant to understand the impact of microbiota on human health and disease.

### Airway Microbiota Dysbiosis in BPD

Contrary to conventional wisdom, the human respiratory tract immediately acquires microbiota, detecting a low bacterial DNA load before or shortly after birth (11–13), which gradually builds up by the 1st month of life, developing colonization of relatively-stable bacteria at the phylum level (14). In this regard, different individuals and even distinct anatomical parts have their unique bacterial colonization patterns, and microbiota in each zone has its preponderant operational taxonomic units (OTUs) (14). Elucidating lung microbiota composition is a challenge, since it requires highly sensitive detection methods and reagents, and collected samples are susceptible to contamination by pathogenic microorganisms in the upper respiratory tract, such as oropharynx and nasopharynx (15). Furthermore, pre- and post-natal lung microbiota is compromised because of the use of antibiotics, maternal chorioamnionitis, mechanical ventilation, infection/inflammation, nutritional deficiencies, and abnormal colonization of the intestine (16). For example, antibiotics enhance the invasive potential of pathogenic bacteria by increasing their nutrient requirements such as organic acids, carbon, and nitrogen, thereby temporarily or permanently reducing the diversity and richness of the microbiota (17). As a result, some advances in research have generated inconsistent results in reporting lung microbiota at birth. However, in general terms, in a period after birth, the pulmonary microbiota composition at the phylum level is predominantly *Proteobacteria* and *Firmicutes* (12, 14).

The incipient composition of the lung microbiota cannot be neglected because it is closely related to the onset of mucosal immunity (18), the development of immune tolerance in the lungs (19), and healthy breathing (15). Several reports have shown alterations in the stability and diversity of the respiratory tract microbiota in BPD. Lohmann et al. (11) tested tracheal aspirates of 25 premature infants at different periods and showed that bacterial multiplicity of 10 patients with BPD significantly decreased, according to the observed species count and the Shannon index. As the disease progresses, *Firmicutes* and *Proteobacteria* populations increase and decrease respectively; at the genus level, the relative abundance of *Acinetobacter sp*. significantly decreases, whereas that of *Staphylococcus* and *Klebsiella* increases. However, microbiota in the non-BPD group invariably maintains high diversity and stability, indicating that lower diversity of airway microbiota may be associated with the disease.

A study by Lal et al. (12) showed that at the phylum level, the *Proteobacteria* amount was higher than that of *Firmicutes* and *Fusobacteria* in babies with BPD, whereas at the genus-level, *Lactobacillus* content was significantly low, which persisted during the disease. The reduction of *Lactobacillus* leading to an inflammatory response and consequently interfering with lung development is an important reason why the microbiota directly affects BPD, as many studies have shown that *Lactobacillus* possesses very strong anti-inflammatory properties. Furthermore, increased endotoxin was observed in the airways of patients with BPD, which was attributed to microbiota dysbiosis.

Lohmann et al. (11) and Lal et al. (12) put forward that conflicting results related to changes in the populations at the phylum level may indicate that lung microbiota is affected by multiple factors, including demographic characteristics, geographic position, living environment, methods and detection reagents, and sequencing platforms. In fact, both studies differed in criteria for the inclusion of children and timing of the sample collection. However, the ecological imbalance of the airway microbiota in children with BPD is an important characteristic in such investigations.

Imamura et al. (20) examined 169 infants with or without severe BPD and noted that all patients with the disease exhibited maladaptive changes in the lung microbiota. The detection rate of *Corynebacterium* species in the lower respiratory tract of severe BPD was higher than that of non-severe disease, and sepsis was commonly observed seven days after birth, speculating that airway microbiota dysbiosis is associated with infections, which may be a significant cause of BPD exacerbation.

Another longitudinal investigation of two research centers found that preterm infants with severe BPD contained abundant *Ureaplasma* after birth, and diversity in lung microbiota was more prominent with age (13). *Ureaplasma* colonization is considered an independent risk factor for BPD (21). On the one hand, it causes chronic infection of the uterine cavity to promote preterm delivery (22), whereas preterm birth is one of the most important causes of BPD. Furthermore, *Ureaplasma* damages the respiratory mucosa and interferes with lung development by producing virulence factors and stimulating the release of pro-inflammatory mediators. Importantly, colonization of the respiratory tract by this species is negatively correlated with gestational age (22), which may partly explain the higher risk of BPD in preterm infants with low gestational age.

In addition, a recent systematic review of microbiota and BPD reported that microbiome disorders are present in patient airways and the frequency of microbiota transformation was associated with BPD impairment (23).

Taken together, lung microbiota composition establishes early in life, but the stabilization and diversity of microorganisms are altered by a number of factors, which are particularly relevant in children with BPD. Therefore, it becomes important to the relationship between microbiome dysregulation and this disease.

### BPD and Lung Microbiota Dysbiosis Association

Although the causal link between lung microbiota and BPD has not been fully demonstrated, it can be assumed that the lung maladaptive microbiome is an effective driver for other potentially harmful factors in this illness (24). Dysregulation of the lung microbiota elicits local or systemic infection/inflammation, activates the immune response (25–27), and is related to oxidative stress and metabolic disorders in the host (24), which impairs dysbiosis, thus creating a vicious circle involved in the disease onset and exacerbation.

#### Gut-lung Axis in BPD

Lung microbiota dysbiosis in BPD may disrupt gut microbiota, which probably plays an important role in worsening this disease. Ryan et al. (28) analyzed stool samples of 50 preterm BPD infants and observed that among transvaginal babies, *Escherichia* and *Shigella* were significantly increased, whereas *Klebsiella* and *Salmonella* were in lower amounts, demonstrating that the disease is involved in gut microbiota dysbiosis. Furthermore, a case-control study of eight subjects with BPD and 10 subjects without the disease showed that gut microbiota diversity in the BPD group was significantly reduced (OTU, relative abundance, and Shannon index), and severe BPD may make gut microbiota more susceptible to destruction early in life (29).

In turn, gut microbiota dysbiosis also affects BPD. Cantey et al. (30) reported that for infants with a very low birth weight receiving antibiotics for 2 weeks significantly increased the risk of death or BPD, which still exists after controlling the severity of the disease. Furthermore, this risk increases with the duration of antibiotic exposure [i.e., every additional day of antibiotics increases the risk of BPD by ~13% (30)]. A possible explanation is that antibiotics destroy the gut microbiota, and the dysbiosis exacerbates the disease. In a perinatal antibiotics-exposed BPD mouse model, it was shown that gut maladaptive microbiota increased pulmonary fibrosis and worsened the condition, which may be related to the reduced expression of lung IL-22 caused by gut microbiota dysbiosis (31). Moreover, gut microbiota, which controls trimethylamine N-oxide (TMAO) production (32), regulates BPD susceptibility by changing TMAO levels (33).

A growing body of research indicates an apparent bidirectional influence of intestinal and lung microbiota. The gut-lung axis hypothesis, which involves a complex cross-talk between lung/gut disease and gut/lung microbiota dysbiosis, has been extensively tested (34–37). Newborn mice that have been depleted of gut microbiota with antibiotics become more susceptible to *Streptococcus pneumoniae* infection, however, restoring intestinal microbiota increases neonatal and germ-free (GF) mice resistance (38) and potentiates phagocytosis of alveolar macrophages (39), thus protecting lungs from bacterial infection. In addition, two separate studies on severe pneumonia and childhood community-acquired pneumonia have shown that maladaptive gut microbiota may be relevant in the onset and development of pneumonia (40, 41). Moreover, the administration of lipopolysaccharide (LPS) in mouse lungs significantly alters gut microbiota (42).

Influenza virus-infected mice develop gut microbiota dysbiosis earlier, which reduces the secretion of metabolites by intestinal microorganisms, thus increasing the susceptibility of mice to *S. pneumoniae* infection (43), suggesting a close link between gut microbiota and the lung immune response. Dickson et al. (44) demonstrated live intestinal bacteria in lungs in a murine model of sepsis and in bronchoalveolar lavage fluid (BALF) of 68 patients with acute respiratory distress syndrome, which indicates that local or systemic inflammation mediates gut microbiota displacement, disrupting the lung microecology homeostasis, and in turn, dysregulated lung microbiota exacerbates the inflammatory response. These data suggest an important interrelationship between gut and lung microbiota, and BPD onset and development. Microbiota dysbiosis in these organs may trigger the inflammatory process, leading to immune disorders and exacerbating the disease outcome. Therefore, preserving the intestines and lungs microecological balance significantly improves BPD.

#### Infection/Inflammation

Infection/inflammation may play a central role in BPD pathogenesis. Stressmann et al. (45) observed a number of pathogens (particularly *S. aureus, Enterobacter* sp., *Moraxella catarrhalis, Pseudomonas aeruginosa*, and *Streptococcus* sp.) in the tracheal secretions of eight preterm infants at risk of developing BPD. Another investigation of 192 newborns found that lung infections in early newborns (particularly in the first 3 days of life) were related to the evolution of chronic lung disease (46). Under a combined effect of other BPD risk factors such as hyperoxia and mechanical ventilation, infection triggers a series of pro-inflammatory substances, such as IL-1β, IL-6, IL-8, NLRP3, TNF-α, and collagen I, which are further regulated by infiltrating neutrophils and macrophages (47–49). These inflammatory mediators in immature lungs of premature infants restrict the activity of surfactant proteins and the vascular endothelial growth factor (49), contributing to the development of alveolar and vascular alterations and other characteristic pathologies in BPD. Furthermore, TLR binding-induced reactive oxygen species (ROS) activate the NLRP3/caspase-1 pathway, which promotes IL-1b production (47), amplifying the inflammation process.

**Figure 2:**
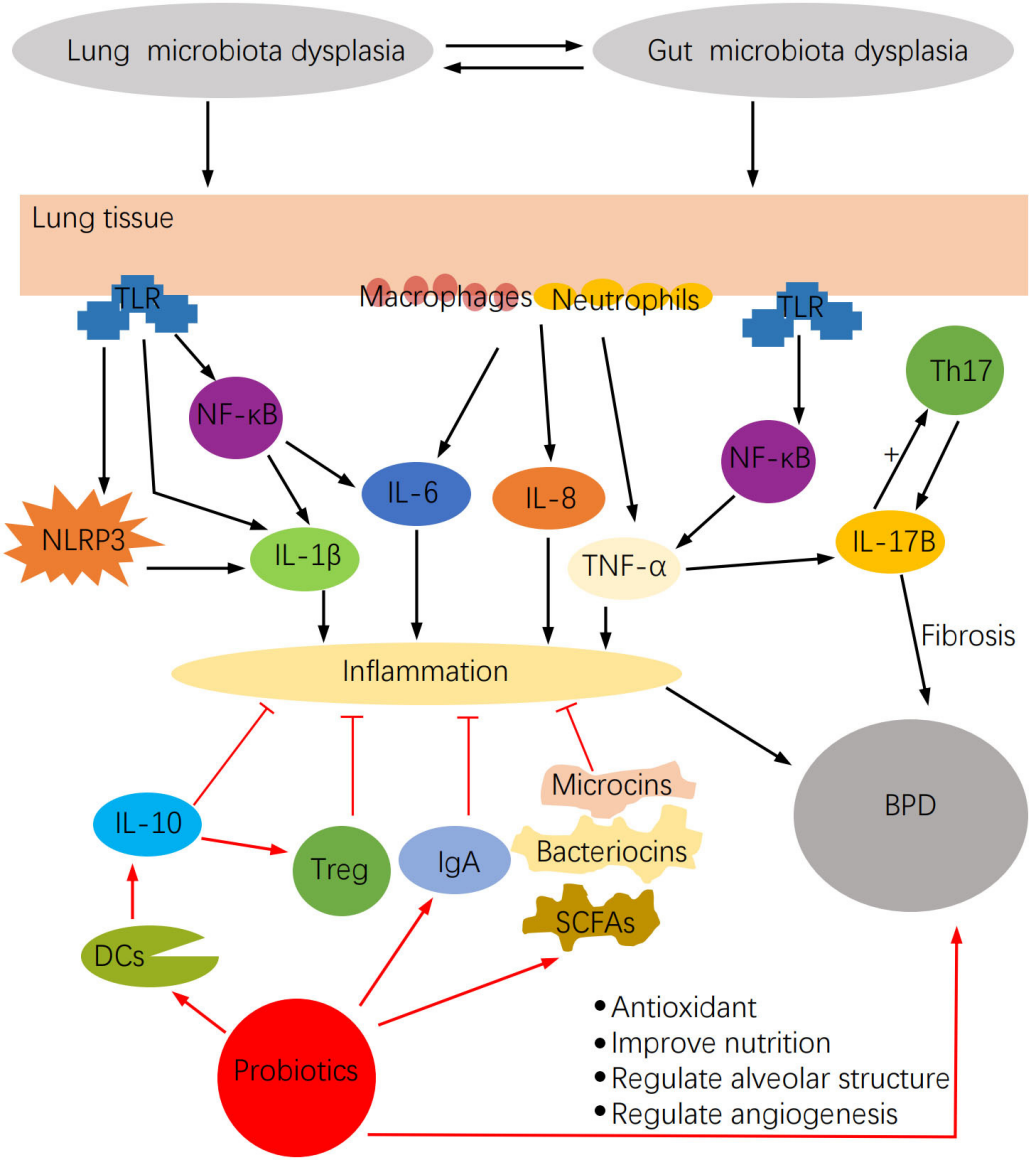
The impact of microbiota dysbiosis on BPD and the potential function of probiotics in BPD.

Microbiota dysbiosis may stimulate a robust inflammatory response and have a significant impact on BPD (Figure 2). Lung microbiota dysbiosis triggers the release of the pro-inflammatory cytokines IL-1β, IL-6, MIP-1α, IL-12p70, and CXCL8, which are associated with pulmonary fibrosis (50). Among them, IL-6 increases with the rise of *Firmicutes* richness, but IL-12P70 augments with the reduction of *Proteobacteria* richness. Similarly, a series of cytokines such as IFN-α2, IL-13, IL-4, IL-15, TNF-α, TNF-β, and MCP1 were detected in the BALF of hematopoietic cell transplantation recipients, and changes in their concentration were mostly associated with lung microbiota dysbiosis (51). In this regard, IFN-α2, IL-13, TNF-β, and TNF-α negatively correlated with the relative abundance of *Firmicutes*, whereas IL-4 and IL-13 positively correlated with *Bacteroidetes*. Another study demonstrated that the increased inflammation observed in the LPS-induced mouse lung injury model was associated with lung microbiota dysbiosis, which enhanced the IL-6 pro-inflammatory effect probably mediated by abnormally dominant OTUs (52). Moreover, in the bleomycin-induced lung fibrosis model, Th1 cells in GF mice were reduced, whereas Foxp3^+^ T regulatory cells were higher compared with non-GF mice, thus demonstrating the regulatory effect of lung microbiota on cellular immunity (50).

**Figure 3:**
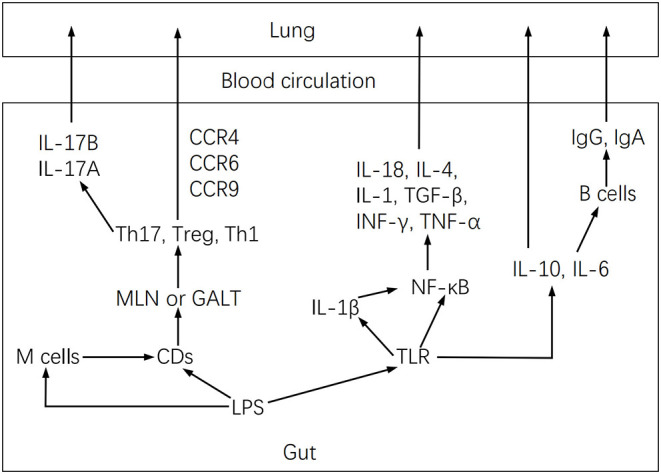
Gut-lung axis diagram.

The inflammatory reaction attributable to the lung maladaptive microbiota may also be closely related to Th17 activation (27). In this regard, the lung microbiota has been shown to modulate local mucosal barrier function by enhancing or reducing the release of IL-17 family cytokines, which are mainly secreted by Th17 cells (26). However, microbiota dysbiosis may activate pulmonary fibrosis by stimulating IL-17B production, which in turn acts on Th17 and neutrophil recruitment genes under TNF-α coordination in a feedback event (53). Furthermore, lung microbiota may intervene in inflammation by altering alveolar macrophages, DCs, invariant natural killer T cells, Treg cells, and lung-resident Tgd cells function (26). These cells induce neutrophil migration. They are involved in the intestine-lung axis by controlling pathogenic microorganisms, maintaining pulmonary homeostasis, and affecting chronic inflammatory activities of lung diseases (26), but microbiota dysbiosis may modify these effects. Dysbiosis activates the lung immune response through the gut-lung axis (Figure 3), by stimulating lymphocyte infiltration in the gut and lung mucosa (54). DCs first recognize pathogens in the intestine and present antigens to T lymphocytes of mesenteric lymph nodes or gut-associated lymphoid tissue, where T cell subsets are activated and migrate to the respiratory mucosa, attracted by chemotactic molecules, and stimulate a local inflammatory response (37). Furthermore, microbiota dysbiosis increases LPS, which stimulates TLR and NF-κB to produce IL-18, IL-4, IL-1, IL-6, TGF-b, IFN-g, and TNF-α involved in pulmonary immune responses (37, 55).

Taken together, these data suggest that gut and lung microbiota may have a significant impact on BPD, and that the disease outcome may be improved by protecting the microbiome.

## Role of Probiotics in BPD

In recent years, the potential of probiotics to exert several biological activities is remarkable. *Lactobacillus reesei* has been shown to be useful in delaying tissue damage and relieving upper respiratory tract infections in patients with cystic fibrosis with mild to moderate lung disease (56), whereas *Bifidobacterium mixture* reduces the clinical manifestations of allergic rhinitis and improves the quality of life of children with the occasional paroxysm of asthma (57). In addition, prophylactic use of probiotics decreases lung infections in mechanically ventilated children (58) and lessens the likelihood of late-onset sepsis in preterm infants (59, 60).

It is not difficult to infer that probiotics have great potential in the treatment of respiratory diseases. More importantly, some studies have shown interest in probiotics and BPD. Consequently, we attempted to explore the feasible influence of probiotics on BPD.

### Anti-infection and Anti-inflammatory Roles of Probiotics

Probiotics, as living microorganisms, become useful in relieving microbiome dysregulations. It has been shown in animal studies that oral probiotics enhance the richness and diversity of airway microbial communities (61), which provide a starting point for understanding the interaction of probiotics and BPD. Furthermore, probiotics have unexpected anti-infection and anti-inflammatory elements and the potential to recover nutrition and antioxidant properties (Figure 2), which are essential for children with BPD.

Oral administration of *Lactobacillus plantarum* was observed to significantly reduce pulmonary inflammation in *Klebsiella pneumoniae*-infected mice, as shown by the decreased number of macrophages and neutrophils, and pro-inflammatory cytokines (KC, IL-6, and TNF-α), and the blocking of NF-κB activation by an interaction with TLR (62). *L. plantarum* also down-regulates T-bet and IL-2 levels, stimulates Foxp3^+^ and z/70mRNA expression in lung tissue, and expands the number of CD4^+^CD25^+^Foxp3^+^ cells in mediastinal lymph nodes (62). Although *L. plantarum* failed to increase the amount of DCs, it attracts them to produce IL-10, promoting Treg cell immunoregulatory action (62).

In a mouse model of *Neisseria meningitides* secondary to influenza A virus (IAV) infection, Belkacem et al. (63) observed that *L. paracasei* increased the amount of DCs, neutrophils, and monocytes in the lungs. Although some highly expressed inflammatory cytokines, such as IL-6, MCP1, KC, and IL-12p70, were detected, only IL-6 and MCP1 were statistically significant. They consider that *L. paracasei* simultaneously augments the health status of IAV and influenza-meningococcal infection in mice, by attracting interstitial monocytes and DCs (63). In addition, intratracheal administration of *Lactobacillus* probably suppresses the PAO1 virulence factor and reduces IL-6 and TNF-α activities, affecting lung infection outcome by *Pseudomonas aeruginosa* in mice (64).

Similarly, *Bifidobacterium bifidum* was shown to produce a significant anti-inflammatory response with a high production of IFN-g, IL-12, and IL-4 (cellular immunity) and IgG1 and IgG2α levels (humoral immunity) in mice infected with influenza virus, but reduced IL-6 production in lung tissue (65). In a mice model of severe asthma, *Bifidobacterium breve* significantly decreased the levels of the pro-inflammatory cytokines IL-1α and IL-1b and the chemokine CXCL-2, which stimulate neutrophil migration to pulmonary tissue, as compared with control, where an increase of IL-1α was observed (66). Furthermore, *B. breve* down-regulated activated CD11b^+^ cells, up-regulated CD4^+^CD44^+^ cells and CD4^+^FoxP3^+^ cells, and increased the number of macrophages.

*In vitro* experiments showed antibacterial activity of *Bifidobacterium* and *Lactobacillus* against *Clostridium* (67), which was probably mediated by bacteriocins, microcins, and short-chain fatty acids (SCFAs) (68). Bacteriocins change the permeability of the inner membrane of pathogenic bacteria, affecting cell wall synthesis (69), whereas microcins directly impair the activity of enzymes required by pathogens in the process of gene replication and transcription (70).

SCFAs generated by probiotics interfere with pathogenicity by reducing intestinal pH, destroying pathogen cell membrane structure, accelerating oxidative phosphorylation, enhancing the antibacterial potential of other molecules (68), regulating the expression of histone deacetylase, and coupling with G protein receptors (71). In addition, SCFAs blocks secretion of IL-8 and macrophage inflammatory protein 2 by intestinal IL-1b in naive mice, evidencing the characteristic anti-inflammatory effect of SCFAs (72). Moreover, probiotics improve immunity by stimulating IgA production through the following mechanisms: (a) triggering the activation of TLR9 and TLR2, (b) promoting DCs maturation, (c) regulating B lymphocytes, (d) being involved in mucosa cytokines production, and (e) inducing the release of TGF-β, IL-10, and IL-6 (68, 71).

### The Antioxidant and Nutritional Effects of Probiotics

It is well-known that oxidative stress plays a central function in BPD pathogenesis. Hyperoxia exposure activates an excessive production of ROS, inhibits the development of alveolar and pulmonary blood vessels, and participates in the inflammatory process (73). In addition, ROS causes endothelial dysfunction and increases vascular permeability, leading to pulmonary tissue edema (74). Microbiota dysbiosis may initiate oxidative stress because lung microbiota stimulates the aryl hydrocarbon receptor, which regulates the activity of antioxidant enzymes, by changing the levels of tryptophan catabolites (74). *Lactobacillus* and *Bifidobacterium* are known to possess antioxidant properties (75). *In vitro* experiments showed that *Lactobacillus plantarum* MA2 inhibits hydrogen peroxide and lipid peroxidation, and possesses the great potential to chelate Fe^2+^ and scavenge free radicals such as 1,1-diphenyl-2-picrylhydrazine (DPPH), hydroxyl radicals, superoxide anion radicals, and has exceptional reducing activity (76). Mechanistically, antioxidant genes (e.g., glutathione peroxidase, catalase, NADH oxidase, NADH peroxidase, and glutathione reductase genes) encode superoxide dismutase and glutathione peroxidase to exert antioxidant effects (76). Furthermore, exopolysaccharides extracted from *Lactobacillus plantarum* possess antioxidant activities. At a concentration of 10 mg/mL, exopolysaccharides reached the maximum 80.4, 65.5, and 60.5% scavenging rates for hydroxyl, 2,2'-azino-bis(3-ethylbenzothiazoline-6-sulfonate), and DPPH radicals, respectively (77). Similarly, *Bifidobacterium* has a high potential to scavenge free radicals and an acceptable reducing activity (78). However, it should be recognized that the antioxidant properties of probiotics are unique. The free radical scavenging activity of probiotics greatly varies from strain to strain (79). In short, probiotics prevent oxidative stress in different ways, ① chelating metal ions, ② activating antioxidant enzymes and metabolites, ③ boosting antioxidant signaling pathways such as Nrf2-Keap1-ARE, mitogen-activated protein kinases, and protein kinase C pathway, ④ reducing ROS production, ⑤ protecting gut microbiota, and ⑥ increasing the host's antioxidant capacity (80).

The nutritional factor is also important in BPD onset. It has been observed that providing enrichment proteins and energy complements to children with protracted mechanical ventilation, reduces the disease incidence (81). Probiotics may increase the nutritional status of preterm infants. In this regard, it was observed that intestinal digestive enzymes (for example, α-amylase, lipase, and trypsin) of mice fed with *Bacillus subtilis* and *Bacillus velezensis* were significantly increased, as compared with control (82). In addition, it was shown that from weeks 2 to 3, the average daily weight gain of mice in the probiotic group was significantly higher and the feed conversion rate was lower, indicating that probiotics promote mice growth. Similarly, probiotics increase the appetite and weight of older dogs, improve immunity, and enrich gut microbiota (83). Moreover, a meta-analysis demonstrated that supplementing with probiotics reduces the time to full enteral feeding for premature infants to achieve better weight gain and growth development (84). Probiotics decrease nutrient wastage by protecting the integrity of the intestinal mucosa, promoting digestion and absorption, and blocking undesirable metabolic pathways (85).

*Lactobacillus sp*. may also alter alveolar structure and regulate alveolar growth (86). Furthermore, probiotics were shown in animal studies to be involved in angiogenesis (87).

In general, given the outstanding advantages of probiotics, we may need to broaden our horizons to fully comprehend the activities and mechanisms of probiotics, particularly associated with their potential to improve BPD outcome.

## Challenge

As we envision the benefits of probiotics for BPD patients, we must also be very aware that there are still numerous problems that must be overcome. A meta-analysis indirectly evaluated the impact of probiotics on BPD (88). Unfortunately, the result was denied by the authors. However, all studies by the authors exclusively use BPD as a secondary result of the research, which may be one of their limitations. In addition, several studies have not given the importance the disease and probiotics deserve, possibly impacting judgment. In fact, the designated species and strains are likely to largely dominate the efficacy of probiotics, which has been certified in tests by Monteiro et al. (67) Different doses and preparations of probiotics may produce divergent conclusions as well. It is also probable that each host system possesses its own “*proprietary probiotics*.”

Detection of high-quality probiotics that may relate to BPD requires a number of well-established, safe, reliable, and laborious scientific methods. However, in any case, this meta-analysis is suggested, as it opens a new direction in the discussion of probiotics and BPD.

For immensely frail infants with BPD, they can only face the following burdensome issues: How to determine the optimal therapy time window and duration of probiotics? Are combination medications used? How to choose the route of administration? Oral administration should anticipate whether probiotics will add to the burden of the gastrointestinal tract and whether a safe dose of probiotics achieves the therapeutic concentration. Theoretically, compared with oral administration, probiotics possibly have a more apparent curative effect by aerosolized inhalation and intratracheal administration, since they directly act on the respiratory mucosa, but this assumption needs experimental verification.

Even though probiotics are beneficial bacteria for humans, they also can produce adverse effects, including involvement in systemic disease, harmful metabolic events, and excessive stimulation of the immune system (89). Thus, it is necessary to find more effective and precise probiotic action pathways to optimize their use in human health. They produce differential effects on the population. Particularly in infancy, premature babies may become a high-risk group to use probiotics, because of their immature immunity. Previous investigations reported that probiotic supplementation early after birth increases the occurrence of mucosal (oral, respiratory, gastrointestinal) infection diseases (90). Infants taking probiotics have complained of unpleasant taste, dry skin, bloating, vomiting, rash, and other adverse events (91). Some premature babies may be affected by bacteremia when receiving probiotics treatment, and the infected strain derives from the probiotics themselves (92, 93). In this regard, it is necessary to conduct a thorough long-term evaluation of the safety of probiotics in children (especially newborns) in consecutive trials, before using them in therapy.

In conclusion, probiotics have promising applications, particularly to improve BPD prognosis. However, it is essential to carefully select the probiotic strains, medication dosage, frequency, and routes. Furthermore, more valid scientific information and follow-up studies are required to support the adequate use of probiotics in human health.

## Author Contributions

KY wrote the manuscript. WD audited the manuscript. All authors contributed to the article and approved the submitted version.

## Conflict of Interest

The authors declare that the research was conducted in the absence of any commercial or financial relationships that could be construed as a potential conflict of interest.

## References

[1] Kalikkot Thekkeveedu R Guaman MC Shivanna B. Bronchopulmonary dysplasia: a review of pathogenesis and pathophysiology. Respir Med. (2017) 132:170–7. 10.1016/j.rmed.2017.10.014 29229093 PMC5729938

[2] Higgins RD Jobe AH Koso-Thomas M Bancalari E Viscardi RM Hartert TV. Bronchopulmonary dysplasia: executive summary of a workshop. J Pediatrics. (2018) 197:300–8. 10.1016/j.jpeds.2018.01.043 29551318 PMC5970962

[3] Hwang JS Rehan VK. Recent advances in bronchopulmonary dysplasia: pathophysiology, prevention, and treatment. Lung. (2018) 196129–38. 10.1007/s00408-018-0084-z 29374791 PMC5856637

[4] Bancalari E Jain D. Bronchopulmonary dysplasia: 50 years after the original description. Neonatology. (2019) 115:384–91. 10.1159/000497422 30974430

[5] Northway WH Rosan RC Porter DY. Pulmonary disease following respiratory therapy of hyaline-membrane disease. N Engl J Med. (1967) 276:357–68. 10.1056/NEJM196702162760701 5334613

[6] Abman SH Bancalari E Jobe A. The evolution of bronchopulmonary dysplasia after 50 years. Am J Respir Crit Care Med. (2017) 195:421–4. 10.1164/rccm.201611-2386ED 28199157

[7] Reid G Jass J Sebulsky MT McCormick JK. Potential uses of probiotics in clinical practice. Clin Microbiol Rev. (2003) 16:658–72. 10.1128/cmr.16.4.658-672.2003 14557292 PMC207122

[8] Lilly DM Stillwell RH. Probiotics: growth-promoting factors produced by microorganisms. Science. (1965) 147:747–8. 10.1126/science.147.3659.747 14242024

[9] Hill C Guarner F Reid G Gibson GR Merenstein DJ Pot B. The international scientific association for probiotics and prebiotics consensus statement on the scope and appropriate use of the term probiotic. Nat Rev Gastroenterol Hepatol. (2014) 11:506–14. 10.1038/nrgastro.2014.66 24912386

[10] Marchesi JR Ravel J. The vocabulary of microbiome research: a proposal. Microbiome. (2015) 3:31. 10.1186/s40168-015-0094-5 26229597 PMC4520061

[11] Lohmann P Luna RA Hollister EB Devaraj S Mistretta TA Welty SE. The airway microbiome of intubated premature infants: characteristics and changes that predict the development of bronchopulmonary dysplasia. Pediatric Res. (2014) 76:294–301. 10.1038/pr.2014.85 24941215

[12] Lal CV Travers C Aghai ZH Eipers P Jilling T Halloran B. The airway microbiome at birth. Sci. Rep. (2016) 6:31023. 10.1038/srep31023 27488092 PMC4973241

[13] Wagner BD Sontag MK Harris JK Miller JI Mourani PM. Airway microbial community turnover differs by BPD severity in ventilated preterm infants. PLoS ONE. (2017) 12:e0170120. 10.1371/journal.pone.0170120 28129336 PMC5271346

[14] Gallacher D Mitchell E Alber D Wach R Klein N Marchesi JR. Dissimilarity of the gut-lung axis and dysbiosis of the lower airways in ventilated preterm infants. Eur Respir J. (2020) 55:1901909. 10.1183/13993003.01909-2019 32060060 PMC7236867

[15] Gallacher DJ Kotecha S. Respiratory microbiome of new-born infants. Front Pediatrics. (2016) 4:10. 10.3389/fped.2016.00010 26942168 PMC4762994

[16] Tirone C Pezza L Paladini A Tana M Aurilia C Lio A. Gut and lung microbiota in preterm infants: immunological modulation and implication in neonatal outcomes. Front Immunol. (2019) 10:2910. 10.3389/fimmu.2019.02910 31921169 PMC6920179

[17] Bäumler AJ Sperandio V. Interactions between the microbiota and pathogenic bacteria in the gut. Nature. (2016) 535:85–93. 10.1038/nature18849 27383983 PMC5114849

[18] Wu BG Segal LN. Lung microbiota and its impact on the mucosal immune phenotype. Microbiol Spectrum. (2017) 5:161–86. 10.1128/microbiolspec.BAD-0005-2016 28643622 PMC5484071

[19] Sommariva M Le Noci V Bianchi F Camelliti S Balsari A Tagliabue E. The lung microbiota: role in maintaining pulmonary immune homeostasis and its implications in cancer development and therapy. Cell Mol Life Sci. (2020) 77:2739–49. 10.1007/s00018-020-03452-8 31974656 PMC7326824

[20] Imamura T Sato M Go H Ogasawara K Kanai Y Maeda H. The microbiome of the lower respiratory tract in premature infants with and without severe bronchopulmonary dysplasia. Am J Perinatol. (2017) 34:80–87. 10.1055/s-0036-1584301 27240094

[21] Viscardi RM Terrin ML Magder LS Davis NL Dulkerian SJ Waites KB. Randomised trial of azithromycin to eradicate *Ureaplasma* in preterm infants. Arch Dis Child Fetal Neonatal Ed. (2020). [Epub ahead of print]. 10.1136/archdischild-2019-318122. 32170033 PMC7592356

[22] Viscardi RM Kallapur SG. Role of ureaplasma respiratory tract colonization in bronchopulmonary dysplasia pathogenesis: current concepts and update. Clin Perinatol. (2015) 42:719–38. 10.1016/j.clp.2015.08.003 26593075 PMC4662049

[23] Pammi M Lal CV Wagner BD Mourani PM Lohmann P Luna RA. Airway microbiome and development of bronchopulmonary dysplasia in preterm infants: a systematic review. J Pediatrics. (2019) 204:126–33.e122. 10.1016/j.jpeds.2018.08.042 30297287

[24] Gentle SJ Lal CV. Predicting BPD: lessons learned from the airway microbiome of preterm infants. Front. Pediatrics. (2019) 7:564. 10.3389/fped.2019.00564 32117822 PMC7011099

[25] Petersen C Round JL. Defining dysbiosis and its influence on host immunity and disease. Cell Microbiol. (2014) 16:1024–33. 10.1111/cmi.12308 24798552 PMC4143175

[26] Yang D Xing Y Song X Qian Y. The impact of lung microbiota dysbiosis on inflammation. Immunology. (2020) 159:156–66. 10.1111/imm.13139 31631335 PMC6954700

[27] Mendez R Banerjee S Bhattacharya SK Banerjee S. Lung inflammation and disease: a perspective on microbial homeostasis and metabolism. IUBMB Life. (2019) 71:152–65. 10.1002/iub.1969 30466159 PMC6352907

[28] Ryan FJ Drew DP Douglas C Leong LEX Moldovan M Lynn M. Changes in the composition of the gut microbiota and the blood transcriptome in preterm infants at <29 weeks gestation diagnosed with bronchopulmonary dysplasia. mSystems. (2019) 4:e00484–19. 10.1128/mSystems.00484-19 31662429 PMC6819732

[29] Chen S-M Lin C-P Jan M-S. Early gut microbiota changes in preterm infants with bronchopulmonary dysplasia: a pilot case-control study. Am J Perinatol. (2020). [Epub ahead of print]. 10.1055/s-0040-1710554. 32446254

[30] Cantey JB Huffman LW Subramanian A Marshall AS Mallett LH Antibiotic exposure and risk for death or bronchopulmonary dysplasia in very low birth weight infants. J Pediatr. (2016) 181:289–93.e1. 10.1016/j.jpeds.2016.11.002 27908652

[31] Willis KA Siefker DT Aziz MM White CT Mussarat N Gomes CK. Perinatal maternal antibiotic exposure augments lung injury in offspring in experimental bronchopulmonary dysplasia. Am J Physiol Lung Cell Mol Physiol. (2020) 318:L407–18. 10.1152/ajplung.00561.2018 31644311 PMC7132329

[32] Wang Z Klipfell E Bennett BJ Koeth R Levison BS Dugar B. Gut flora metabolism of phosphatidylcholine promotes cardiovascular disease. Nature. (2011) 472:57–63. 10.1038/nature09922 21475195 PMC3086762

[33] Piersigilli F Bhandari V. Metabolomics of bronchopulmonary dysplasia. Clin Chim Acta Int J Clin Chem. (2020) 500:109–14. 10.1016/j.cca.2019.09.025 31689411

[34] Piersigilli F Van Grambezen B Hocq C Danhaive O. Nutrients and microbiota in lung diseases of prematurity: the placenta-gut-lung triangle. Nutrients. (2020) 12:469. 10.3390/nu12020469 32069822 PMC7071142

[35] Wypych TP Wickramasinghe LC Marsland BJ. The influence of the microbiome on respiratory health. Nat Immunol. (2019) 20:1279–90. 10.1038/s41590-019-0451-9 31501577

[36] Anand S Mande SS. Diet, microbiota and gut-lung connection. Front Microbiol. (2018) 9:2147. 10.3389/fmicb.2018.02147 30283410 PMC6156521

[37] Samuelson DR Welsh DA Shellito JE. Regulation of lung immunity and host defense by the intestinal microbiota. Front Microbiol. (2015) 6:1085. 10.3389/fmicb.2015.01085 26500629 PMC4595839

[38] Gray J Oehrle K Worthen G Alenghat T Whitsett J Deshmukh H. Intestinal commensal bacteria mediate lung mucosal immunity and promote resistance of newborn mice to infection. Sci Transl Med. (2017) 9:eaaf9412. 10.1126/scitranslmed.aaf9412 28179507 PMC5880204

[39] Schuijt TJ Lankelma JM Scicluna BP De Sousa E Melo F Roelofs JJ. The gut microbiota plays a protective role in the host defence against pneumococcal pneumonia. Gut. (2016) 65:575–83. 10.1136/gutjnl-2015-309728 26511795 PMC4819612

[40] Zhang X Yang X Zhang Z Lei M Zhang X Wang X. Analysis of intestinal patients' flora changes with severe pneumonia based on 16SrDNA sequencing technology. Chin Crit Care Med. (2019) 31:1479–84. 10.3760/cma.j.issn.2095-4352.2019.12.009 32029033

[41] Ren X Gamallat Y Liu D Zhu Y Meyiah A Yan C. The distribution characteristics of intestinal microbiota in children with community-acquired pneumonia under five years of age. Microbial Pathog. (2020) 142:104062. 10.1016/j.micpath.2020.104062 32058024

[42] Sze MA Tsuruta M Yang SW Oh Y Man SF Hogg JC. Changes in the bacterial microbiota in gut, blood, and lungs following acute LPS instillation into mice lungs. PLoS ONE. (2014) 9:e111228. 10.1371/journal.pone.0111228 25333938 PMC4205020

[43] Sencio V Barthelemy A Tavares LP Machado MG Soulard D Cuinat C. Gut dysbiosis during influenza contributes to pulmonary pneumococcal superinfection through altered short-chain fatty acid production. Cell Rep. (2020) 30:2934. 10.1016/j.celrep.2020.02.013 32130898

[44] Dickson RP Singer BH Newstead MW Falkowski NR Erb-Downward JR Standiford TJ. Enrichment of the lung microbiome with gut bacteria in sepsis and the acute respiratory distress syndrome. Nat Microbiol. (2016) 1:16113. 10.1038/nmicrobiol.2016.113 27670109 PMC5076472

[45] Stressmann FA Connett GJ Goss K Kollamparambil TG Patel N Payne MS. The use of culture-independent tools to characterize bacteria in endo-tracheal aspirates from pre-term infants at risk of bronchopulmonary dysplasia. J Perinatal Med. (2010) 38:333–7. 10.1515/JPM.2010.026 20121490

[46] Beeton ML Maxwell NC Davies PL Nuttall D McGreal E Chakraborty M. Role of pulmonary infection in the development of chronic lung disease of prematurity. Eur Respir J. (2011) 37:1424–30. 10.1183/09031936.00037810 20884745

[47] Papagianis PC Pillow JJ Moss TJ. Bronchopulmonary dysplasia: pathophysiology and potential anti-inflammatory therapies. Paediatr Respir Rev. (2019) 30:34–41. 10.1016/j.prrv.2018.07.007 30201135

[48] Savani RC. Modulators of inflammation in bronchopulmonary dysplasia. Semin Perinatol. (2018) 42:459–70. 10.1053/j.semperi.2018.09.009 30446300 PMC6368974

[49] Pan J Zhan C Yuan T Wang W Shen Y Sun Y. Effects and molecular mechanisms of intrauterine infection/inflammation on lung development. Respir Res. (2018) 19:93. 10.1186/s12931-018-0787-y 29747649 PMC5946538

[50] O'Dwyer DN Ashley SL Gurczynski SJ Xia M Wilke C Falkowski NR. Lung microbiota contribute to pulmonary inflammation and disease progression in pulmonary fibrosis. Am. J. Respir. Crit. Care Me. (2019) 199:1127–38. 10.1164/rccm.201809-1650OC 30789747 PMC6515865

[51] O'Dwyer DN Zhou X Wilke CA Xia M Falkowski NR Norman KC. Lung dysbiosis, inflammation, and injury in hematopoietic cell transplantation. Am J Respir Crit Care Med. (2018) 198:1312–21. 10.1164/rccm.201712-2456OC 29878854 PMC6290939

[52] Poroyko V Meng F Meliton A Afonyushkin T Ulanov A Semenyuk E. Alterations of lung microbiota in a mouse model of LPS-induced lung injury. Am J Physiol Lung Cell Mol Physiol. (2015) 309:L76–83. 10.1152/ajplung.00061.2014 25957290 PMC4491514

[53] Yang D Chen X Wang J Lou Q Lou Y Li L. Dysregulated lung commensal bacteria drive interleukin-17B production to promote pulmonary fibrosis through their outer membrane vesicles. Immunity. (2019) 50:692–706.e697. 10.1016/j.immuni.2019.02.001 30824326

[54] Forsythe P. Probiotics and lung immune responses. Ann Am Thoracic Soc. (2014) 11:S33–7. 10.1513/AnnalsATS.201306-156MG 24437403

[55] Kou H Fu Y He Y Jiang J Gao X Zhao H. Chronic lead exposure induces histopathological damage, microbiota dysbiosis and immune disorder in the cecum of female Japanese quails (*Coturnix japonica*). Ecotoxicol Environ Saf. (2019) 183:109588. 10.1016/j.ecoenv.2019.109588 31450035

[56] Di Nardo G Oliva S Menichella A Pistelli R De Biase RV Patriarchi F.. *Lactobacillus reuteri* ATCC55730 in cystic fibrosis. J Pediatric Gastroenterol Nutr. (2014) 58:81–86. 10.1097/MPG.0000000000000187 24121143

[57] Miraglia Del Giudice M Indolfi C Capasso M Maiello N Decimo F Ciprandi G. Bifidobacterium mixture (B longum BB536, B infantis M-63, B breve M-16V) treatment in children with seasonal allergic rhinitis and intermittent asthma. Ital J Pediatrics. (2017) 43:25. 10.1186/s13052-017-0340-5 28270216 PMC5341466

[58] Banupriya B Biswal N Srinivasaraghavan R Narayanan P Mandal J Probiotic prophylaxis to prevent ventilator associated pneumonia (VAP) in children on mechanical ventilation: an open-label randomized controlled trial. Intensive Care Med. (2015) 41:677–85. 10.1007/s00134-015-3694-4 25708419

[59] Zhang G-Q Hu H-J Liu C-Y Shakya S Li Z-Y. Probiotics for preventing late-onset sepsis in preterm neonates: a PRISMA-compliant systematic review and meta-analysis of randomized controlled trials. Medicine. (2016) 95:e2581. 10.1097/MD.0000000000002581 26937897 PMC4778994

[60] Rao SC Athalye-Jape GK Deshpande GC Simmer KN Patole SK. Probiotic supplementation and late-onset sepsis in preterm infants: a meta-analysis. Pediatrics. (2016) 137:e20153684. 10.1542/peds.2015-3684 26908700

[61] Vientos-Plotts AI Ericsson AC Rindt H Reinero CR. Oral probiotics alter healthy feline respiratory microbiota. Front Microbiol. (2017) 8:1287. 10.3389/fmicb.2017.01287 28744273 PMC5504723

[62] Vareille-Delarbre M Miquel S Garcin S Bertran T Balestrino D Evrard B. Immunomodulatory effects of *Lactobacillus plantarum* on inflammatory response induced by Klebsiella pneumoniae. Infect Immun. (2019) 87:e00570–19. 10.1128/IAI.00570-19 31481408 PMC6803346

[63] Belkacem N Bourdet-Sicard R Taha M-K. Lactobacillus paracasei feeding improves the control of secondary experimental meningococcal infection in flu-infected mice. BMC Infect Dis. (2018) 18:167. 10.1186/s12879-018-3086-9 29636018 PMC5894232

[64] Fangous MS Alexandre Y Hymery N Gouriou S Arzur D Blay GL. Lactobacilli intra-tracheal administration protects from pulmonary infection in mice - a proof of concept. Beneficial Microbes. (2019) 10:893–900. 10.3920/BM2019.0069 31965833

[65] Mahooti M Abdolalipour E Salehzadeh A Mohebbi SR Gorji A Ghaemi A. Immunomodulatory and prophylactic effects of bifidobacterium bifidum probiotic strain on influenza infection in mice. World J Microbiol Biotechnol. (2019) 35:91. 10.1007/s11274-019-2667-0 31161259

[66] Raftis EJ Delday MI Cowie P McCluskey SM Singh MD Ettorre A. Bifidobacterium breve MRx0004 protects against airway inflammation in a severe asthma model by suppressing both neutrophil and eosinophil lung infiltration. Sci Rep. (2018) 8:12024. 10.1038/s41598-018-30448-z 30104645 PMC6089914

[67] Monteiro C Do Carmo MS Melo BO Alves MS Dos Santos CI Monteiro SG. *in vitro* antimicrobial activity and probiotic potential of bifidobacterium and lactobacillus against species of clostridium. Nutrients. (2019) 11:448. 10.3390/nu11020448 30795551 PMC6412307

[68] Do Carmo MS Santos CID Araujo MC Giron JA Fernandes ES Monteiro-Neto V. Probiotics, mechanisms of action, and clinical perspectives for diarrhea management in children. Food Funct. (2018) 9:5074–95. 10.1039/c8fo00376a 30183037

[69] Wiedemann I Böttiger T Bonelli RR Schneider T Sahl H-G Martínez B. Lipid II-based antimicrobial activity of the *Lantibiotic plantaricin C*. Appl Environ Microbiol. (2006) 72:2809–14. 10.1128/AEM.72.4.2809-2814.2006 16597986 PMC1449081

[70] Duquesne S Destoumieux-Garzón D Peduzzi J Rebuffat S. Microcins, gene-encoded antibacterial peptides from Enterobacteria. Nat Product Rep. (2007) 24:708–34. 10.1039/B516237H 17653356

[71] Hardy H Harris J Lyon E Beal J Foey AD. Probiotics, prebiotics and immunomodulation of gut mucosal defences: homeostasis and immunopathology. Nutrients. (2013) 5:1869–912. 10.3390/nu5061869 23760057 PMC3725482

[72] Zheng N Gao Y Zhu W Meng D Walker WA. Short chain fatty acids produced by colonizing intestinal commensal bacterial interaction with expressed breast milk are anti-inflammatory in human immature enterocytes. PLoS ONE. (2020) 15:e0229283. 10.1371/journal.pone.0229283 32084202 PMC7034856

[73] Wang J Dong W. Oxidative stress and bronchopulmonary dysplasia. Gene. (2018) 678:177–83. 10.1016/j.gene.2018.08.031 30098433

[74] Capasso L Vento G Loddo C Tirone C Iavarone F Raimondi F. Oxidative stress and bronchopulmonary dysplasia: evidences from microbiomics, metabolomics, and proteomics. Front Pediatrics. (2019) 7:30. 10.3389/fped.2019.00030 30815432 PMC6381008

[75] Mishra V Shah C Mokashe N Chavan R Yadav H Prajapati J. Probiotics as potential antioxidants: a systematic review. J Agric Food Chem. (2015) 63:3615–26. 10.1021/jf506326t 25808285

[76] Tang W Xing Z Li C Wang J Wang Y. Molecular mechanisms and *in vitro* antioxidant effects of *Lactobacillus plantarum* MA2. Food Chem. (2017) 221:1642–9. 10.1016/j.foodchem.2016.10.124 27979141

[77] Min W-H Fang X-B Wu T Fang L Liu C-L Wang J. Characterization and antioxidant activity of an acidic exopolysaccharide from *Lactobacillus plantarum* JLAU103. J Biosci Bioeng. (2019) 127:758–66. 10.1016/j.jbiosc.2018.12.004 30600152

[78] Wang B-G Xu H-B Xu F Zeng Z-L Wei H. Efficacy of oral bifidobacterium bifidum ATCC 29521 on microflora and antioxidant in mice. Can J Microbiol. (2016) 62:249–62. 10.1139/cjm-2015-0685 26863255

[79] Amaretti A di Nunzio M Pompei A Raimondi S Rossi M Bordoni A. Antioxidant properties of potentially probiotic bacteria: *in vitro* and *in vivo* activities. Appl Microbiol Biotechnol. (2013) 97:809–17. 10.1007/s00253-012-4241-7 22790540

[80] Wang Y Wu Y Wang Y Xu H Mei X Yu D Antioxidant properties of probiotic bacteria. Nutrients. (2017) 9:521 10.3390/nu9050521 PMC5452251 28534820

[81] Klevebro S Westin V Stoltz Sjostrom E Norman M Domellof M Edstedt Bonamy AK. Early energy and protein intakes and associations with growth, BPD, and ROP in extremely preterm infants. Clin Nutr. (2019) 38:1289–95. 10.1016/j.clnu.2018.05.012 29885776

[82] Li A Wang Y Li Z Qamar H Mehmood K Zhang L. Probiotics isolated from yaks improves the growth performance, antioxidant activity, and cytokines related to immunity and inflammation in mice. Microbial Cell Fact. (2019) 18:112. 10.1186/s12934-019-1161-6 31217027 PMC6585042

[83] Xu H Huang W Hou Q Kwok L-Y Laga W Wang Y. Oral administration of compound probiotics improved canine feed intake, weight gain, immunity and intestinal microbiota. Front Immunol. (2019) 10:666. 10.3389/fimmu.2019.00666 31001271 PMC6454072

[84] Athalye-Jape G Deshpande G Rao S Patole S. Benefits of probiotics on enteral nutrition in preterm neonates: a systematic review. Am J Clin Nutr. (2014) 100:1508–19. 10.3945/ajcn.114.092551 25411286

[85] Judkins TC Archer DL Kramer DC Solch RJ. Probiotics, nutrition, and the small intestine. Curr Gastroenterol Rep. (2020) 22:1–8. 10.1007/s11894-019-0740-3 31930437

[86] Yun Y Srinivas G Kuenzel S Linnenbrink M Alnahas S Bruce KD. Environmentally determined differences in the murine lung microbiota and their relation to alveolar architecture. PLoS ONE. (2014) 9:e113466. 10.1371/journal.pone.0113466 25470730 PMC4254600

[87] Chen X Yang G Song J-H Xu H Li D Goldsmith J. Probiotic yeast inhibits VEGFR signaling and angiogenesis in intestinal inflammation. PLoS ONE. (2013) 8:e64227. 10.1371/journal.pone.0064227 23675530 PMC3652827

[88] Villamor-Martinez E Pierro M Cavallaro G Mosca F Kramer B Villamor E Probiotic supplementation in preterm infants does not affect the risk of bronchopulmonary dysplasia: a meta-analysis of randomized controlled trials. Nutrients. (2017) 9:1197 10.3390/nu9111197 PMC5707669 29088103

[89] Marteau P Safety aspects of probiotic products. Näringsforskning. (2001) 45:22–24. 10.3402/fnr.v45i0.1785

[90] Quin C Estaki M Vollman DM Barnett JA Gill SK Gibson DL. Probiotic supplementation and associated infant gut microbiome and health: a cautionary retrospective clinical comparison. Sci Rep. (2018) 8:8283. 10.1038/s41598-018-26423-3 29844409 PMC5974413

[91] Sotoudegan F Daniali M Hassani S Nikfar S Abdollahi M. Reappraisal of probiotics' safety in human. Food Chem Toxicol. (2019) 129:22–29. 10.1016/j.fct.2019.04.032 31009735

[92] Bertelli C Pillonel T Torregrossa A Prod'hom G Fischer CJ Greub G. *Bifidobacterium longum* bacteremia in preterm infants receiving probiotics. Clin Infect Dis. (2015) 60:924–7. 10.1093/cid/ciu946 25472946

[93] Esaiassen E Cavanagh P Hjerde E Simonsen GS Støen R Klingenberg C. *Bifidobacterium longum* subspecies infantis bacteremia in 3 extremely preterm infants receiving probiotics. Emerg Infect Dis. (2016) 22:1664–6. 10.3201/eid2209.16003 27532215 PMC4994345

